# 

**Published:** 1916-12-02

**Authors:** 


### CONTENTS.

PAGE

**The Treatment of Lunatics** ... ... ... 173

**Report of the Medical Research Committee...** 174

**Hospital and Institutional News** ... ... ... 175

Death of Sir George White—The Sinking of Hospital Ships—Brownlow Hill Infirmary—The Late Dr. Doyen—The Late Mr. Charles Booth—A Sword for a Fund's Vice-Chairman—Orthopædic Surgery for the Wounded—A Wittenberg Memorial—An X-Ray Victim at Middlesex Hospital—A New Test for Enteric Fevers—Sir Hiram Maxim and a Hospital Secretary—A Benefactor of the Mount Vernon Hospital—Beds to be Doubled at Nottingham—Paying Patients in Dundee—The Extension of Harrogate Infirmary—Ellen Terry at the Royal Eye Hospital, Southwark—A Doctor's Effective Invention for Soldiers—Enormous Contingent Bequests for London Hospitals and Charities—Camp Sanitation for Medical Recruits—The Dispensary Chain in London—Strange Attempted Suicide—A Lancashire Superintendent on the Mentally Defective Child—Spurring the Public Conscience at Plymouth—The Devonshire Hospital, Buxton—The Care of Discharged Soldiers at Birmingham—A Soldier's Gift to Soldiers—A Telegraphic Signal System—This Week's Drug Market.

**The Hospital Dietary** ... ... ... ... 181

A Too Much Neglected Study.

**The School Doctor** ... ... ... ... ... ... 183

The School Doctor and Sex Education.

PAGE

**Natural History Notes from Macedonia** ... 184

Some Observations of Bird Life.

**The Institutional Library** ... ... ... ... 185

Human Temperaments—Life Saving in War-time—Our Hospital A B C—The Midwife's Pronouncing Dictionary.

**The Matrons' and Sisters' Department** ... 187

The College of Nursing: A Call to Action.

Round the Hospitals.

An Indefatigable President—The Women of the United States—Zoos in Schools—Scottish Nurses and Registration—An Example to be Followed.

**The Modified Pay System** ... ... ... ... 189

Patients' Payments and Contributions.

**The Editor's Letter-Box** ... ... ... ... 191

Asylum Workers.

**Gifts to Hospitals** ... ... ... .. ... 191

**Parliament and the Profession** ... ... ... 192

**Matrons' and Sisters' Appointments** ... ... 192

**Personalia**... ... ... ... ... ... ... ... 192

**The Institutional Worker** ... ... (Suppl.) 1

War Conditions and Hospital Expenditure.
Stationery Stocks in an Institution.
Question for December.
Enquiries and Answers.

